# Nephrotic Syndrome as an Extramuscular Manifestation of Anti-EJ Antibody-Positive Dermatomyositis: A Case Report and Review of the Literature

**DOI:** 10.1155/2022/1233522

**Published:** 2022-09-20

**Authors:** Syoko Tsubouchi, Takahiro Mizuuchi, Yusuke Yamamoto, Daiki Fujimori, Kayo Ishii, Mayu Tago, Eri Kato, Hiroaki Mori, Haeru Hayashi, Koichiro Tahara, Tetsuji Sawada

**Affiliations:** Department of Rheumatology, Tokyo Medical University Hospital, Shinjuku, Tokyo, Japan

## Abstract

Renal involvement is underestimated as an extramuscular manifestation of dermatomyositis (DM). Here, we describe a 67-year-old woman with anti-glycyl-transfer ribonucleic acid synthetase (anti-EJ) antibody and anti-ribonucleoprotein antibody-positive DM complicated by systemic sclerosis, who developed nephrotic syndrome concurrently with the exacerbation of DM, as indicated by incremental serum creatine kinase levels, high-intensity lesions on muscle magnetic resonance imaging, and active interstitial pneumonitis on chest computed tomography. Renal biopsy revealed the presence of immune-deposition in the glomerulus by immunofluorescence. To our knowledge, this is the first report describing the coexistence of anti-EJ antibody-positive DM and nephrotic syndrome. More reports of similar cases are warranted to substantiate the association.

## 1. Introduction

Polymyositis (PM) and dermatomyositis (DM) are two main types of autoimmune inflammatory muscle disease and are clinically characterized by symmetrical weakness and myalgia of proximal muscles with elevated serum levels of muscle enzymes [1]. DM is characterized by distinctive skin manifestations, including Gottron's sign, Gottron's papules, heliotrope rash, and mechanic's hands. Autoimmune abnormalities manifest as the presence of autoantibodies, which can be categorized into myositis-associated and myositis-specific autoantibodies. Anti-aminoacyl-transfer ribonucleic acid (tRNA) synthetase antibodies (anti-ARS Abs) are classified as myositis-specific autoantibodies and are detected in PM and DM patients presenting with myositis, non-erosive polyarthritis, Raynaud's phenomenon, mechanic's hands, and interstitial pneumonitis, designated as anti-synthetase syndrome (ASS) [2]. The characteristics of each anti-ARS Ab vary slightly in terms of incidence and the clinical course of interstitial lung disease, skin rashes, myopathies, and arthritis [3, 4]. Anti-glycyl tRNA synthetase antibodies (anti-EJ Abs) are the second most prevalent anti-ARS Ab in Japan after anti-Jo1 Ab, accounting for approximately a quarter of ASS cases, and are associated with classic DM with a high frequency of interstitial lung disease (ILD) [4].

Renal involvement, which is frequently observed in patients with systemic autoimmune diseases such as systemic lupus erythematosus (SLE), has been considered rare in PM and DM. However, case reports describing the simultaneous occurrence of PM or DM and renal diseases have been reported in the literature [5]. Two main types of renal involvement exist in patients with PM or DM: rhabdomyolysis-induced acute tubular necrosis with deterioration of renal function and chronic glomerulonephritis [6]. In the latter, mesangial proliferative glomerulonephritis is the leading histological type in PM, while membranous nephropathy appears to prevail in DM [6]. Herein, we describe the case of a woman who developed nephrotic syndrome concurrently with the exacerbation of anti-EJ Ab-positive DM with active interstitial pneumonitis. To our knowledge, this is the first description of anti-EJ Ab-positive DM associated with nephrotic syndrome.

## 2. Case

We describe here the case of a 67-year-old Japanese woman with a 30-year history of DM and a 2-year history of limited cutaneous systemic sclerosis (SSc), who developed nephrotic syndrome that occurred concurrently with the exacerbation of DM.

She developed Raynaud's phenomenon when she was 27 years old. In terms of DM, she developed Gottron's papules, a heliotrope rash, polyarthralgia, and proximal muscle weakness with elevated muscle enzymes at the age of 37. She was diagnosed with DM and treated with corticosteroids. Although the full details of the disease and treatment course were unavailable, she had been treated with a maintenance dose of 10 mg prednisolone daily and 4 mg methotrexate (MTX) weekly, when she developed bilateral hand contracture and subsequent digital ulcers at the age of 66. At this point, the doses of prednisolone and MTX were increased to 15 mg per day and 8 mg per week, respectively, and further increased, 2 months later, to 30 mg per day and 12 mg per week, respectively. Despite the strengthened treatment, the digital ulcers exacerbated, and she was admitted to our hospital, when she was 67 years old (first admission). It was revealed that the case had been complicated by bacterial tenosynovitis refractory to antibiotic treatment, which required amputation of the right middle finger at the base of the proximal phalanx. The digital ulcers were ameliorated by vasodilator treatments, including sympathetic blocks and the administration of calcium antagonists. During the perioperative period, weekly MTX was discontinued, and the dose of prednisolone was reduced over a couple of months to 10 mg per day, when she was discharged from the hospital.

**Figure 1:**
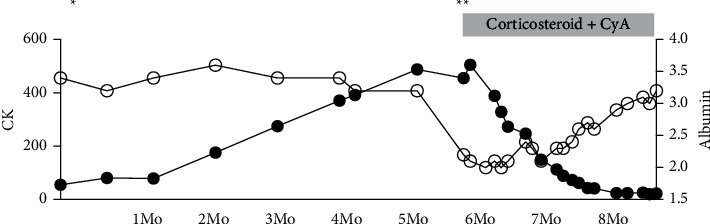
Graph showing serum levels of creatine kinase (CK) and albumin (Alb) over time after discharge from first admission to our hospital. The left vertical axis indicates the serum CK levels ((U/L), closed black circles), and the right axis indicates serum albumin (Alb) levels ((mg/dL), open circles). The horizontal line indicates the months (Mo) after discharge from first admission. ^*∗*^indicates the timing of discharge of first admission to our hospital. ^*∗∗*^indicates readmission to our hospital. CyA: cyclosporine A.

The digital ulcers did not recur during the follow-up period after hospital discharge. However, 3 months after hospital discharge, the patient presented with mechanic's hands and proximal muscle pain and weakness, with elevated serum creatine kinase (CK) levels. The serum CK levels continued to increase over several months (Figure 1), at which point she developed hypoalbuminemia and nephrotic-range proteinuria, and was admitted to our hospital again.

**Figure 2:**
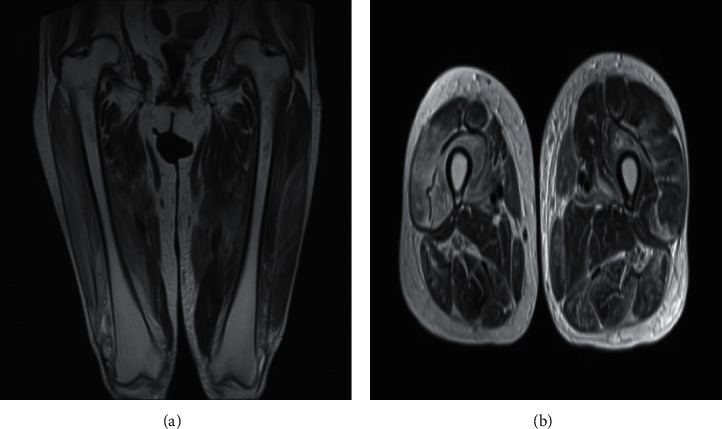
Magnetic resonance imaging (MRI) of bilateral thigh muscles of a woman who developed recurrent anti-glycyl tRNA synthetase (EJ) antibody-positive dermatomyositis concurrently with newly developed nephrotic syndrome shown are sagittal (a) and axial. (b) MRI images demonstrating wide-spread high-intensity lesions on T2-weighted sequences.

On second admission, the patient measured 149 cm in height and weighed 49.3 kg. Her vital signs were as follows: temperature of 37.5°C, pulse rate of 80 beats per minute, blood pressure of 101/89 mmHg, and oxygen saturation (SpO_2_) measured by pulse oximetry of 99% on room air and at rest. The palpebral conjunctiva was not anemic, and the bulbar conjunctiva was not icteric. Cardiac sounds were normal. Fine crackles were heard during late inspiration at the posterior base of both lungs. Abdominal examination revealed no abnormalities. The skin on the ulnar sides of the thumbs and radial sides of the index fingers appeared hyperkeratotic and cracked, compatible with mechanic's hands. No heliotrope rash or Gottron's papules were noted. There was tenderness over her upper arms and thighs bilaterally. Mild pretibial pitting edema was noted in both legs. The laboratory findings are shown in Table 1. Of note, serum levels of muscle-derived enzymes including CK, aspartate aminotransferase, lactate dehydrogenase, myoglobulin, and aldolase were elevated. Muscle magnetic resonance imaging (MRI) revealed the presence of diffuse high signal intensity lesions in the bilateral thigh on T2-weighted and short-tau inversion recovery (STIR) sequences (Figure2). Immunological examination showed positive antinuclear antibodies at a titer of 1 : 320 with speckled patterns and 1 : 40 with cytoplasmic patterns (normal value, <1 : 40). Anti-ARS Ab was positive at an index of 174 (normal value, <5.0 index), and anti-EJ Ab was subsequently demonstrated to be positive by immunoblot analysis. Anti-ribonucleoprotein (RNP) Ab, anti-anti-Sjögren's syndrome A (SSA) Ab, and anti-anti-Sjögren's syndrome B (SS-B) Ab were also positive, but anti-double-stranded deoxyribonucleic acid (dsDNA) Ab, anti-Smith (Sm) Ab, anti-phospholipid Ab, anti-myeloperoxidase (MPO) Ab, and anti-proteinase-3 (PR3) Ab were absent. Electrocardiography showed no abnormalities. Serum Krebs von den Lungen-6 (KL-6) was elevated at 1163 U/mL (normal value, <500 U/mL), and chest computed tomography (CT) showed reticular shadows preferentially at the periphery of the lower lungs bilaterally, indicating the presence of active interstitial pneumonitis. Regarding musculoskeletal symptoms, she was diagnosed with exacerbation of dermatomyositis complicated by interstitial pneumonitis after reducing immunosuppressive treatments.

**Figure 3:**
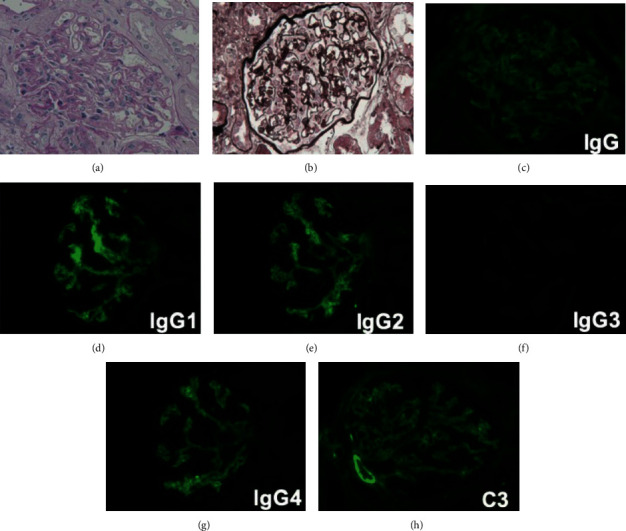
Renal biopsy showing deposition of immunoglobulin G (IgG) and complement 3 (C3) in a woman who developed recurrent anti-glycyl tRNA synthetase (EJ) antibody-positive dermatomyositis concurrently with newly developed nephrotic syndrome. (a) Hematoxylin and eosin (HE) stain; (b) periodic acid methenamine silver (PAM) stain; immunofluorescence examination: (c) total IgG; (d) IgG1; (e) IgG2; (f) IgG3; (g) IgG4; (h) C3. Light microscopic examinations using HE and PAM stains show neither thickening nor spike formation of basement membranes. Deposition of IgG, IgG1, IgG2, IgG4, and C3 is noted. Immunoglobulin A (IgA) and immunoglobulin M (IgM) are absent (data not shown).

As for renal injuries, urinalysis showed 3+ proteinuria, with 1–4 red blood cells per high power field (HPF), 1–4 white blood cells per HPF, >100 hyaline casts per HPF, 1–4 epithelial casts per HPF, and 1–4 fatty casts per HPF, in urinary sediment analysis. The urine protein-to-creatinine ratio was 8.09, and urinary protein excretion was 3.97 g per day. Together with low serum albumin levels (2.0 g/dL) and elevated levels of total cholesterol (295 mg/dL), she was diagnosed with nephrotic syndrome. To determine the underlying renal pathology of nephrotic syndrome, a renal biopsy was performed (Figure 3). Light microscopic examination using hematoxylin-eosin, periodic acid Schiff, Masson's trichrome, and periodic acid methenamine silver staining showed neither thickening nor spike formation of basement membranes. However, the deposition of immunoglobulin G (IgG) (++), IgG1 (+++), IgG2 (+++), IgG3 (−), IgG4 (+++), immunoglobulin A (IgA) (−), immunoglobulin M (IgM) (±), complement 3 (C3) (+++), complement 1q (C1q) (±), and complement 4 (C4) (−) was observed in the glomerulus by immunofluorescence (Figure 3), which suggests the possibility of membranous nephropathy at an early stage, i.e., before the deposition of immune complexes causes apparent basement thickening.

**Figure 4:**
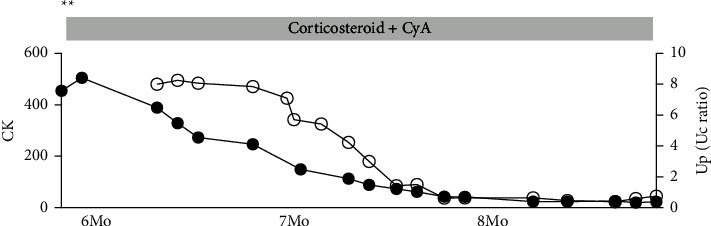
Graph showing serum levels of creatine kinase (CK) and urine protein-to-creatinine ratio (Up/Uc ratio) over time after second admission to our hospital. The left vertical axis indicates the serum CK levels ((U/L), closed black circles), and the right axis indicates the degree of proteinuria, expressed as (Up/Uc) ratio (open circles). The horizontal line indicates the months (Mo) after discharge of first admission. Up: urinary protein; Uc: urinary creatinine. ^*∗∗*^indicates readmission to our hospital. CyA: cyclosporine A.

Collectively, the diagnosis of nephrotic syndrome concurrently with exacerbation of anti-EJ Ab-positive DM with interstitial pneumonitis was made. The dose of prednisolone was increased from 10 mg to 40 mg daily, and cyclosporine was initiated at a dose of 100 mg daily, which 2 weeks later was increased to 150 mg daily, together with the anti-platelet drug dipyridamole. This led to normalization of serum CK levels and proteinuria expressed as urine protein-to-creatinine ratio (Figure 4). The dose of prednisolone was gradually decreased thereafter over 6 months to a maintenance dose of 5 mg daily without recurrence.

## 3. Discussion

In the present study, we describe the case of a woman who presented with nephrotic syndrome concurrent with the recurrence of DM. Renal damage is considered rare as an extramuscular manifestation of PM and DM [1]. However, it has recently been postulated that renal involvement is not as uncommon as previously thought [7]. Yen et al. retrospectively investigated the complications of renal disease in 65 patients with PM or DM over a 10-year period and demonstrated that 14 patients (21.5%) were found to show varying degrees of renal involvement, which were attributed to two disease categories: acute tubular necrosis and chronic glomerulonephritis [8]. Recently, Couvrat-Desvergnes et al. also performed a retrospective study involving 150 patients with inflammatory myopathies, including DM, PM, and ASS [5], with a 4.1-year follow-up period, and demonstrated that renal involvement was present in 35 patients (23.3%) who were categorized into acute kidney injury (*n* = 16) and chronic kidney disease (CKD) (*n* = 31), including those who shifted from AKI to CKD.

Acute kidney injury related to PM or DM can develop during the acute phases of rhabdomyolysis, leading to myoglobinuria, which causes acute tubular necrosis and subsequent renal failure. Patients with PM or DM often have high serum CK levels, usually 10-fold and sometimes up to 50-fold the upper limit of normal values [9, 10]. It was previously considered that rhabdomyolysis leading to acute renal failure was uncommon in PM and DM because the rate and degree of myolysis could be lower than those of myotoxic drug-related necrotizing myopathy [11]. However, myoglobinuria-induced acute tubular necrosis caused by PM or DM has been reported in the literature, especially in patients with DM [12–20]. The other type of renal damage related to PM or DM is chronic glomerulonephritis, which is presumed to be immune-mediated, and exhibits a variety of histologic features. From the 65 PM and DM patients reported by Yen et al., a renal biopsy was performed in two DM patients with overt proteinuria and membranous nephropathy was revealed in one and IgA nephropathy in the other [8]. As for the study by Couvrat-Desvergnes et al., they identified 14 patients who had undergone renal biopsy (6 PM, 5 DM, and 3 ASS), which revealed the presence of glomerulonephritis with various pathologic patterns including immune complex-related glomerulonephritis (*n* = 4), membranous nephropathy (*n* = 2), and IgA nephropathy (*n* = 2) [5]. We conducted an additional literature search and retrieved case reports describing the association of PM or DM with a variety of pathological glomerular lesions, including mesangial proliferative glomerulonephritis [21–25], membranous nephropathy [26–31], membranoproliferative glomerulonephritis [32], IgA nephropathy [33–37], diffuse proliferative glomerulonephritis [38], immune complex-type glomerulonephritis [39–42], acute interstitial nephritis [43], lipoid nephrosis and focal glomerulosclerosis [44], focal segmental glomerulosclerosis with evidence of acute tubular injury [45], sclerotic GN with fibrocellular crescent [46], and crescentic glomerulonephritis associated with malignancy [47]. Cases presenting with anti-neutrophil cytoplasmic antibody-associated glomerulonephritis in the context of DM have also been reported [48–50]. As pointed out in previous studies [6, 25], there is a tendency for mesangial proliferative glomerulonephritis to be more frequently observed in PM, while membranous nephropathy is more frequently observed in the context of DM.

The present case was positive for anti-ARS Ab, which was measured using MESACUP anti-ARS tests (MBL, Japan) that detected five anti-ARS Abs (anti-Jo-1, anti-PL-7, anti-PL-12, anti-EJ, and anti-KS Abs). The patient was subsequently found to have anti-EJ Ab using BlueDiver immunoblot kits (D-tek, Belgium) that measured each anti-ARS Ab separately. The frequency of anti-EJ Ab in ASS varies among previous studies, ranging from 1.8% to 23% [4, 51–53]. Thus, Pinal-Fernandez et al. investigated the frequency of anti-ARS Abs based on the Johns Hopkins Myositis Center cohort and demonstrated that anti-Jo1 Ab was most prevalent (73.4%) in ASS, followed by anti-PL12 (13.6%), anti-PL7 (9.5%), anti-EJ (1.8%), and anti-OJ (1.8%) Abs [51]. Based on the AENEAS (American and European Network of anti-synthetase syndrome) collaborative group's cohort database, Cavagna et al. also showed that anti-EJ Ab was the fourth anti-ARS Ab with its frequency of 4.5% (38 out of 828 patients with ASS) [52]. Furthermore, Hamaguchi et al. reported that anti-EJ Ab was detected in 38 out of 166 Japanese patients with ASS (23%) [4]. As for its frequency in China, Zhan et al. demonstrated that anti-EJ Ab was the third most prevalent anti-ARS Ab, preceded by anti-Jo-1 and anti-PL7 Abs in patients with ASS complicated by ILD with its frequency of 21% (23 out of 108 patients) [53].

To our knowledge, this is the first case report describing the association of anti-EJ Ab with nephrotic syndrome. It should be noted here that our patient, who developed nephrotic syndrome concurrently with the recurrence of DM, was positive for anti-RNP Ab and anti-SSB Ab in addition to anti-EJ Ab. Regarding the involvement of anti-SSB Abs, they are immunological markers for Sjögren's syndrome (SS) [54]. Although the renal manifestation of SS is usually type I distal renal tubular acidosis and tubular interstitial nephritis, glomerulonephritis can be found, albeit less frequently [55]. Therefore, the possibility cannot be excluded that nephrotic syndrome developed in relation to SS. However, we consider that the synchronicity of the development of nephrotic syndrome with the exacerbation of DM supports the association with DM in our patient. As for anti-RNP Ab, this can be detected alone in various autoimmune diseases (mixed connective tissue disease (MCTD), rheumatoid arthritis (RA), SS, SSc, and inflammatory myositis) or together with anti-Sm Ab specifically in systemic lupus erythematosus [56]. Although dermatomyositis overlapping with lupus nephritis has been described in the literature [57–59], our patient showed neither anti-Sm Ab, anti-dsDNA IgG Ab, anti-phospholipid Ab, or hypocomplementemia, thereby making the diagnosis of SLE less likely.

In our case, Raynaud's phenomenon and skin changes at the fingertips were so severe that the distal ends of the phalanges were denuded. Since the overlapping features of SSc, SLE, and PM or DM in MCTD are usually mild with favorable outcomes [60], the patient was considered to have SSc rather than MCTD. The simultaneous detection of anti-ARS Ab and anti-RNP Ab is relatively uncommon. Hamaguchi et al. examined the clinical features of 166 adult patients with anti-ARS Abs, including anti-histidyl tRNA synthetase (anti-Jo-1) Ab (*n* = 59), anti-EJ Ab (*n* = 38), anti-threonyl tRNA synthetase (anti-PL-7) Ab (*n* = 29), anti-alanyl tRNA synthetase (anti-PL-12) Ab (*n* = 18), anti-asparaginyl tRNA synthetase (anti-KS) Ab (*n* = 13), anti-isoleucyl tRNA synthetase (anti-OJ) Ab (*n* = 8), anti-PL7/PL12 Ab (*n* = 1), and demonstrated that anti-RNP Ab was detected in only 2 patients, including one with anti-PL7 Ab and another with anti-PL12 Ab [4]. Previous case-based studies described the patients with anti-RNP Ab coexisting anti-Jo1 Ab [61–64]. To our knowledge, this is the first report describing a patient positive with both anti-EJ Ab and anti-RNP Ab who presented with DM and nephrotic syndrome.

The patient in the present case developed nephrotic syndrome at a time when the disease activity of DM had been increasing. The temporal concurrence of nephrotic syndrome and DM suggests that the two diseases share a common pathophysiological process in this patient. Of course, it should be noted that the simultaneous occurrence of nephrotic syndrome and DM could have been a coincidence. Of interest, Fukui et al. previously used immunohistochemical methods to analyze the components of immune complexes deposited in the glomerulus in a patient with DM that was associated with immune complex (IC)-type membranous nephropathy. It was demonstrated that renal tubular epithelial (RTE) antigens were present specifically at the granular IC disposition sites, suggesting that RTE antigens could be autoantigens in this case. Although their findings have not been replicated subsequently, it would be interesting to search for autoantigens in our patients using modern proteomic technologies. It should also be noted here that the present case was positive for anti-RNP Ab, as described elsewhere. Casal-Dominguez et al. have recently demonstrated that anti-RNP Ab was related to glomerulonephritis in patients with inflammatory myositis [64], in which the clinical phenotypes of 21 patients with anti-RNP Ab-positive myositis were compared to those of 178 patients with DM, 135 patients with immune-mediated necrotizing myopathy, and 132 patients with ASS. They demonstrated that arthritis (60%), DM-specific skin features (60%), and interstitial lung disease (45%) were the most common extramuscular clinical features of anti-RNP Ab-positive myositis. Notably, glomerulonephritis and pericarditis were less common but uniquely found during the disease course in 25% and 40% of patients with anti-RNP Ab-positive myositis, respectively. Further accumulation of cases with anti-EJ Ab-positive dermatomyositis complicated by nephrotic syndrome in the presence or absence of anti-RNP Ab is warranted.

A limitation of this case report is that we did not perform electromyography or muscle biopsy for the evaluation of inflammatory myositis. However, the patient presented with symmetrical proximal muscle weakness and pain, increased serum CK levels, and muscle signal on MRI, together with rashes typical of DM. Furthermore, she was positive for anti-EJ Ab specific for DM [65]. Collectively, we diagnosed her with overlap of dermatomyositis and SSc rather than MCTD. Another limitation was that we did not measure anti-M-type phospholipase A2 receptor antibody, which has been demonstrated to be associated with primary membranous nephropathy [66–68].

In conclusion, we have described a woman with limited cutaneous SSc who developed nephrotic syndrome in the setting of the recurrence of anti-EJ Ab-positive DM. The association of membranous nephropathy with anti-Jo1 Ab-positive DM has been described in the literature. However, to the best of our knowledge, the association of nephrotic syndrome with anti-EJ Ab-positive DM is described for the first time in the present case report. We believe that this report is a valuable addition to the literature since the renal manifestations of PM and DM remain to be firmly established. Further studies investigating patients with DM complicated by nephrotic syndrome according to category of different patterns of autoantibodies, such as myositis-specific and myositis-related Abs, are warranted to better understand the renal manifestations of DM.

## Figures and Tables

**Table 1:** Laboratory values on second admission of a woman with limited cutaneous systemic sclerosis who developed nephrotic syndrome in the setting of recurrence of anti-glycyl-transfer ribonucleic acid synthetase antibody (anti-EJ)-positive dermatomyositis.

Laboratory test	Result	Reference range
*CBC*		
WBC count (/*μ*L)	10,500	2,700–8,800
Lymphocyte (%)	16.6	19–47
RBC count (/*μ*L)	458	370–540
Hemoglobin (g/dL)	12.6	11.0–17.0
Hematocrit (%)	39.0	34.0–49.0
Platelets (×10^4^/*μ*L)	40.8	14–34

*Coagulation*		
PT-INR	1.12	
APTT (sec)	33.1	30.0 ± 5.0

*Biochemistry*		
Total protein (g/dL)	7.0	6.6–8.2
Albumin (g/dL)	2.0	3.9–4.9
AST (U/L)	38	8–38
ALT (U/L)	27	4–44
LDH (U/L)	440	106–211
*γ*GTP (U/L)	9	16–73
Alkaline phosphatase (U/L)	191	104–338
Total bilirubin (mg/dL)	0.50	0.2–1.2
BUN (mg/dL)	17.3	8.0–22.6
Creatinine (mg/dL)	0.56	0.4–0.8
CK (U/L)	1163	43–165
ESR (mm/hour)	19.0	<15
CRP (mg/dL)	2.09	<0.3
KL-6 (U/mL)	1163	<430
SP-A (ng/mL)	124.2	<43.8
SP-D (ng/mL)	202	<110

*Urinalysis*		
Proteinuria	≥300 mg/dL	(−)
WBC (/HPF)	1–4	<1–4
RBC (/HPF)	1–4	<1–4
Casts (/HPF)	Hyaline casts ≥ 100 Epithelial casts 1–4 Fatty casts 1–4	(−)

*Immunological*		
IgG (mg/dL)	2,623	870–1,700
IgA (mg/dL)	412	110–410
IgM (mg/dL)	273	35–220
CH50 (U/mL)	47	25–50
C3 (mg/dL)	80	65–135
C4 (mg/dL)	15	13–35
ANA	1 : 320 speckled 1 : 40 cytoplasmic	<1 : 40
Anti‐dsDNA IgG Ab (IU/mL)	<10	<12
Anti‐SSA Ab	Positive at a titer of 1 : 16	Negative
Anti‐SSB Ab	Positive at a titer of 1 : 2	Negative
Anti‐RNP Ab	Positive at a titer of 1 : 16	Negative
Anti‐Sm Ab	Negative	Negative
Anti-ARS Ab (U/mL)	174.0	<25.0
Anti-EJ Ab	Positive	Negative
LAC	0.83	≤1.16
Ant-cardiolipin Ab (U/mL)	≤8	<10
Anti-*β*2GPI Ab (U/mL)	≤1.2	<3.5
PR3-ANCA (U/mL)	<1.0	<3.5
MPO-ANCA (U/mL)	<1.0	<3.5

CBC: complete blood count; WBC: white blood cell; RBC: red blood cell; PT-INR: prothrombin time-international normalized ratio; APTT: activated partial thromboplastin time; sec: seconds; AST: aspartate aminotransferase; ALT: alanine aminotransferase; LDH: lactate dehydrogenase; *γ*GTP: *γ*glutamyl transpeptidase; BUN: blood urea nitrogen; CK; creatine kinase; ESR: erythrocyte sedimentation rate; CRP: C-reactive protein; KL-6: krebs von den lungen-6; SP: surfactant protein; HPF: high power field; IgG; immunoglobulin G; IgM; immunoglobulin M; IgA; immunoglobulin A; CH50; 50% hemolytic complement activity; C3: complement 3; C4: complement 4; ANA: antinuclear antibody; dsDNA: double-stranded deoxyribonucleic acid; Ab: antibody; SSA: Sjögren's syndrome-related antigen A; SSB: Sjögren's syndrome type B; RNP: ribonucleoprotein; Sm: Smith; ARS: aminoacyl-transfer ribonucleic acid (tRNA) synthetase; EJ: glycyl-transfer ribonucleic acid synthetase; LAC: lupus anticoagulant; *β*2GPI: *β*2-glycoprotein; PR3: proteinase 3; MPO: myeloperoxidase; ANCA: anti-neutrophil cytoplasmic antibody.

## Data Availability

Data can be available on adequate request.

## References

[1] Ascherman D., Aggarwal R., Oddis C., Hochberg M., Gravallese E., Silman A., Smolen J., Weinblatt M., Weisman M. (2019). Classification, epidemiology, and clinical features of inflammatory muscle disease. *Rheumatology*.

[2] Marco J. L., Collins B. F. (2020). Clinical manifestations and treatment of antisynthetase syndrome. *Best Practice & Research Clinical Rheumatology*.

[3] Mammen A. L., Allenbach Y., Stenzel W. (2020). 239th ENMC international workshop: classification of dermatomyositis, Amsterdam, Netherlands. *Neuromuscular Disorders*.

[4] Hamaguchi Y., Fujimoto M., Matsushita T. (2013). Common and distinct clinical features in adult patients with anti-aminoacyl-tRNA synthetase antibodies: heterogeneity within the syndrome. *PLoS One*.

[5] Couvrat-Desvergnes G., Masseau A., Benveniste O. (2014). The spectrum of renal involvement in patients with inflammatory myopathies. *Medicine (Baltimore)*.

[6] Kronbichler A., Mayer G. (2013). Renal involvement in autoimmune connective tissue diseases. *BMC Medicine*.

[7] Cucchiari D., Angelini C. (2017). Renal involvement in idiopathic inflammatory myopathies. *Clinical Reviews in Allergy and Immunology*.

[8] Yen T. H., Lai P. C., Chen C. C., Hsueh S., Huang J. Y. (2004). Renal involvement in patients with polymyositis and dermatomyositis. *International Journal of Clinical Practice*.

[9] Dalakas M. C. (2015). Inflammatory muscle diseases. *New England Journal of Medicine*.

[10] Jones J., Wortmann R. (2015). Idiopathic inflammatory myopathies-a review. *Clinical Rheumatology*.

[11] Rider L. G., Miller F. W. (1995). Laboratory evaluation of the inflammatory myopathies. *Clinical and Diagnostic Laboratory Immunology*.

[12] Kessler E., Weinberger I., Rosenfeld J. B. (1972). Myoglobinuric acute renal failure in a case of dermatomyositis. *Israel Journal of Medical Sciences*.

[13] Singhal P. C., Narayanan-Nampoory M. R., Visweswaran R. K., Mehta R. L., Gupta V., Chugh K. S. (1985). Myoglobinuric renal failure associated with dermatomyositis. *Journal of the Association of Physicians of India*.

[14] Park S. K., Cho K. H., Kang S. K. (1990). Acute renal failure associated with dermatomyositis and colon cancer. *Nephron*.

[15] Caccamo D. V., Keene C. Y., Durham J., Peven D. (1993). Fulminant rhabdomyolysis in a patient with dermatomyositis. *Neurology*.

[16] Lazar A. I. (1994). Acute myositis complicated by myoglobinuric acute renal failure. *Journal of the Association of Physicians of India*.

[17] Rose M. R., Kissel J. T., Bickley L. S., Griggs R. C. (1996). Sustained myoglobinuria: the presenting manifestation of dermatomyositis. *Neurology*.

[18] Joshi D., Kumar N., Rai A. (2009). Dermatomyositis presenting with rhabdomyolysis and acute renal failure; an uncommon manifestation. *Annals of Indian Academy of Neurology*.

[19] Sabha M. M., Simo H. T., Shadid R. M., Altorok N. I. (2018). Successful treatment of antisynthetase syndrome presenting as rhabdomyolysis with rituximab. *Rheumatology International*.

[20] Hamadeh M., Boustany S., Fares J. (2019). Acute kidney injury following dermatomyositis. *Clinical Medicine and Research*.

[21] Dyck R. F., Katz A., Gordon D. A. (1979). Glomerulonephritis associated with polymyositis. *Journal of Rheumatology*.

[22] Frost N. A., Morand E. F., Hall C. L., Maddison P. J., Bhalla A. K. (1993). Idiopathic polymyositis complicated by arthritis and mesangial proliferative glomerulonephritis: case report and review of the literature. *Rheumatology*.

[23] Picco P., Gattomo M., Barbano G. C., Trivelli A., Pistoia V., Buoncompagni A. (1997). Mesangial glomerulonephritis and transient SLE manifestations in an adolescent with dermatomyositis. *Lupus*.

[24] Valenzuela O. F., Reiser I. W., Porush J. G. (2001). Idiopathic polymyositis and glomerulonephritis. *Journal of Nephrology*.

[25] Takizawa Y., Kanda H., Sato K. (2007). Polymyositis associated with focal mesangial proliferative glomerulonephritis with depositions of immune complexes. *Clinical Rheumatology*.

[26] Hara I., Kurata N., Hyozu K., Tamao H. (1991). A case of polymyositis complicated with membranous nephritis. *Nihon Naika Gakkai Zasshi*.

[27] Makino H., Hirata K., Matsuda M., Amano T., Ota Z. (1994). Membranous nephropathy developing during the course of dermatomyositis. *Journal of Rheumatology*.

[28] Rose J. D. (1979). Membranous glomerulonephritis, dermatomyositis, and bronchial carcinoma. *BMJ*.

[29] Soylu A., Kavukçu S., Türkmen M., Saroğlu S. (2001). Dermatomyositis with membranous nephropathy. *Turkish Journal of Pediatrics*.

[30] Akashi Y., Inoh M., Gamo N. (2002). Dermatomyositis associated with membranous nephropathy in a 43-year-old female. *American Journal of Nephrology*.

[31] Hanaoka M., Tsutsumi H., Sano T. (1999). A case of membranous nephropathy associated with polymyositis. *Kitasato Medicine*.

[32] Owlia M. B., Hemayati R., Taghipour Zahir S., Moeini Nodeh M. (2012). Dermatomyositis sine myositis with membranoproliferative glomerulonephritis. *Case Reports in Rheumatology*.

[33] Vilppula A. H., Aine R. A. T. (1984). Polymyositis associated with several immunological disorders. *Clinical Rheumatology*.

[34] Yen T. H., Huang J. Y., Chen C. Y. (2003). Unexpected IgA nephropathy during the treatment of a young woman with idiopathic dermatomyositis: case report and review of the literature. *Journal of Nephrology*.

[35] Civilibal M., Selcuk Duru N., Ozagari A., Durali K., Elevli M. (2009). Immunoglobulin A nephropathy associated with juvenile dermatomyositis. *Pediatric Nephrology*.

[36] Barros T. B. M., Souza F. H. C., Malheiros D. M. A. C., Levy-Neto M., Shinjo S. K. (2014). IgA nephropathy and polymyositis: a rare association. *Revista Brasileira de Reumatologia*.

[37] Mantoo M. R., Tripathy S. K., Phulware R. H., Bagri N. K., Hari P., Barwad A. (2019). Juvenile dermatomyositis with IgA nephropathy: case-based review. *Rheumatology International*.

[38] Xie Q., Liu Y., Liu G., Yang N., Yin G. (2010). Diffuse proliferative glomerulonephritis associated with dermatomyositis with nephrotic syndrome. *Rheumatology International*.

[39] Moriyama T., Uruta Y., Yamaguchi H. (1989). A case of immune-complex type glomerulonephritis associated with dermatomyositis. *Nihon Naika Gakkai Zasshi*.

[40] Fukui H., Kimura T., Nakabayashi K., Nagasawa T. (1976). Dermatomyositis associated with immune-complex type nephritis induced by tubular epithelial antigen. *Nihon Jinzo Gakkai Shi*.

[41] Kaneoka H., Sasatomi Y., Miyagi K. (2003). A rare case of dermatomyositis associated with immune-complex type glomerulonephritis, idiopathic thrombopenic purpura, pulmonary fibrosis and lung cancer. *Clinical & Experimental Rheumatology*.

[42] Kamata K., Kobayashi Y., Shigematsu H., Saito T. (1982). Childhood type polymyositis and rapidly progressive glomerulonephritis. *Pathology International*.

[43] Das J., George P., Pawar B., Calton N. (2008). Acute interstitial nephritis in association with polymyositis. *Journal of Postgraduate Medicine*.

[44] Moutsopoulos H., Fye K. H. (1975). Letter: lipoid nephrosis and focal glomerulosclerosis in a patient with polymyositis. *The Lancet*.

[45] Nickavar A., Mehr Azma M. (2012). Nephrotic syndrome and juvenile dermatomyositis. *Rheumatology International*.

[46] Tsunemi M., Ishimura E., Tsumura K. (1993). A case of crescentic glomerulonephritis associated with polymyositis. *Nephron*.

[47] Chiu K. C., Tsai T. C., Lin W. T., Lee W. J., Su C. C., Chen C. Y. (2008). Paraneoplastic polymyositis associated with crescentic glomerulonephritis. *Renal Failure*.

[48] Kawai H., Kitagawa W., Suzuki N. (2011). Myeloperoxidase-antineutrophil cytoplasmic antibody-related crescentic glomerulonephritis after treatment for clinically amyopathic dermatomyositis: a coincidental combination or not?. *Clinical and Experimental Nephrology*.

[49] Yuste C., Rapalai M., Pritchard B. A. (2014). Overlap between dermatomyositis and ANCA vasculitides. *Clinical Kidney Journal*.

[50] Panda P. K., Suri T. M., Sood R., Bhalla A. S., Sharma M. C., Ranjan P. (2017). Overlap syndrome: juvenile dermatomyositis and perinuclear antineutrophil cytoplasmic autoantibody vasculitis, a case report and review of literature. *International Journal of Rheumatic Diseases*.

[51] Pinal-Fernandez I., Casal-Dominguez M., Huapaya J. A. (2017). A longitudinal cohort study of the anti-synthetase syndrome: increased severity of interstitial lung disease in black patients and patients with anti-PL7 and anti-PL12 autoantibodies. *Rheumatology*.

[52] Cavagna L., Trallero-Araguás E., Meloni F. (2019). Influence of antisynthetase antibodies specificities on antisynthetase syndrome clinical spectrum time course. *Journal of Clinical Medicine*.

[53] Zhan X., Yan W., Wang Y. (2021). Clinical features of anti-synthetase syndrome associated interstitial lung disease: a retrospective cohort in China. *BMC Pulmonary Medicine*.

[54] Feist E., Burmester G., Hochberg M., Gravallese E., Silman A., Smolen J., Weinblatt M., Weisman M. (2019). Laboratory tests in rheumatic disorders. *Rheumatology*.

[55] Goules A., Geetha D., Arend L. J., Baer A. N. (2019). Renal involvement in primary Sjögren’s syndrome: natural history and treatment outcome. *Clinical & Experimental Rheumatology*.

[56] Quismorio F., Quismorio A., Wallace D., Hahn B. (2019). Clinical application of serologic tests, serum protein abnormalities, and other clinical laboratory tests in systemic lupus erythematosus. *Dubois’ Lupus Erythematosus and Related Syndromes*.

[57] Ristic G., Paunić Z., Vojvodić D., Petronijević M., Glisić B., Stefanović D. (2005). Systemic lupus erythematosus and dermatomyositis--case report. *Srpski Arhiv Za Celokupno Lekarstvo*.

[58] Nakaya I., Iwata Y., Sugiyama Y., Abe T., Nomura G. (2002). Dermatomyositis relapse complicated with gastric carcinoma and lupus nephritis five years after the initial diagnosis of dermatomyositis. *Internal Medicine*.

[59] Bitencourt N., Solow E. B., Wright T., Bermas B. L. (2020). Inflammatory myositis in systemic lupus erythematosus. *Lupus*.

[60] Reiseter S., Gunnarsson R., Corander J. (2017). Disease evolution in mixed connective tissue disease: results from a long-term nationwide prospective cohort study. *Arthritis Research and Therapy*.

[61] Lundberg I., Nennesmo I., Hedfors E. (1992). A clinical, serological, and histopathological study of myositis patients with and without anti-RNP antibodies. *Seminars in Arthritis and Rheumatism*.

[62] Brouwer R., Hengstman G. J., Vree Egberts W. (2001). Autoantibody profiles in the sera of European patients with myositis. *Annals of the Rheumatic Diseases*.

[63] Feist E., Schneider M. L., Brychcy M., Dörner T., Burmester G. R., Hiepe F. (2003). A unique autoantibody pattern of positive anti-Jo-1, anti-U1RNP, and antiproteasome antibodies in autoimmune myositis as a diagnostic challenge. *Annals of the Rheumatic Diseases*.

[64] Casal-Dominguez M., Pinal-Fernandez I., Corse A. M. (2019). Muscular and extramuscular features of myositis patients with anti-U1-RNP autoantibodies. *Neurology*.

[65] Alenzi F. M. (2020). Myositis specific autoantibodies: a clinical perspective. *Open Access Rheumatology Research and Reviews*.

[66] Akiyama S., Akiyama M., Imai E., Ozaki T., Matsuo S., Maruyama S. (2015). Prevalence of anti-phospholipase A2 receptor antibodies in Japanese patients with membranous nephropathy. *Clinical and Experimental Nephrology*.

[67] Hayashi N., Akiyama S., Okuyama H. (2015). Clinicopathological characteristics of M-type phospholipase A2 receptor (PLA2R)-related membranous nephropathy in Japanese. *Clinical and Experimental Nephrology*.

[68] Katsumata Y., Okamoto Y., Moriyama T. (2020). Clinical usefulness of anti-M-type phospholipase-A-receptor antibodies in patients with membranous nephropathy and the comparison of three quantification methods. *Immunological Medicine*.

